# A Comparative Review of Fertility and Semen Assessment Techniques in Farm Animals

**DOI:** 10.3390/ani16050854

**Published:** 2026-03-09

**Authors:** Nada N. A. M. Hassanine, Nasir A. Ibrahim, Nosiba S. Basher, Ahmed A. Saleh, Shaaban S. Elnesr, Mohamed Osman Abdalrahem Essa, Hosameldeen Mohamed Husien, Mengzhi Wang

**Affiliations:** 1 College of Animal Science and Technology, Yangzhou University, Yangzhou 225009, China; dh25034@stu.yzu.edu.cn (N.N.A.M.H.); elemlak1339@yzu.edu.cn (A.A.S.); dh23054@stu.yzu.edu.cn (M.O.A.E.); 008643@yzu.edu.cn (H.M.H.); 2 Department of Biology, College of Science, Imam Mohammad Ibn Saud Islamic University (IMSIU), Riyadh 11623, Saudi Arabia; nsbasher@imamu.edu.sa; 3 Animal and Fish Production Department, Faculty of Agriculture (Al-Shatby), Alexandria University, Alexandria City 11865, Egypt; 4 Department of Poultry Production, Faculty of Agriculture, Fayoum University, Fayoum 63514, Egypt; ssn00@fayoum.edu.eg

**Keywords:** CASA, fertility, semen assessment, MTT, farm animals

## Abstract

This review provides a comparative examination of current methods for evaluating fertility and semen quality in mammals, with emphasis on livestock species. It is based on a structured search and analysis of 316 academic publications retrieved from major scientific databases, including Elsevier, Springer, Taylor & Francis, and MDPI, as well as other repositories, covering literature published between 1938 and 2025. This review examines advanced semen analysis techniques, including computer-assisted semen analysis (CASA) systems and assays for assessing chromatin integrity, DNA damage, seminal plasma composition, sperm membrane proteins, and functional parameters such as migration capacity, membrane integrity, acrosomal status, capacitation, morphology, viability, concentration, motility, and volume. Methods for semen collection are also outlined. The synthesis focuses on the comparative assessment of techniques and recent findings for major ruminant and monogastric livestock species: cattle (*Bos taurus* and *Bos indicus*), buffalo (*Bubalus bubalis*), sheep (*Ovis aries*), goats (*Capra hircus*), and pigs (*Sus domesticus*). A brief historical overview of assisted insemination is presented, incorporating relevant procedures and managerial factors that affect fertility outcomes. Advances in genetics related to reproductive health are also discussed. This synthesis seeks to integrate existing knowledge and guide future research in reproductive science for both animal production and human medicine.

## Simple Summary

This review systematically compares techniques for assessing fertility in livestock, drawing on 316 scientific publications. It traces the development from foundational methods to modern technologies for semen analysis and female fertility evaluation, outlining their critical role in improving animal reproduction and clinical andrology.

## 1. Introduction

Fertility is a cornerstone of profitable livestock production, determining herd output and underpinning sustainable agriculture. Successful breeding management requires a reliable understanding of fertility assessment. This review examines established and emerging techniques for evaluating fertility, with a primary focus on selected livestock species of high global economic importance: cattle, buffalo, sheep, goats, and pigs. Where instructive comparisons are drawn to foundational human clinical andrology studies. The strengths and limitations of techniques are discussed within this defined zoological context [1,2,3,4,5].

Puberty onset and early sexual development involve complex interactions between genetic and environmental factors, with breed, climate, nutrition, and genetic strains significantly influencing pubertal timing [6,7,8,9,10]. Further complexity arises from divergent pathways of male and female sexual maturation, rendering a unified definition of puberty challenging due to the multiple biological processes involved [11,12,13,14,15]. As mentioned by Hafez [16], puberty represents the developmental stage when increasing gonadotropic activity from the brain synchronizes with the gonads’ capacity for steroidogenesis and gametogenesis.

The consequences of pubertal timing are directly relevant to livestock management. In sheep production, for instance, lambs that reach puberty earlier and display intense sexual activity contribute to higher overall flock fertility within a breeding season, which in turn allows for more rapid genetic improvement. In rams, successful reproduction depends on hormones such as testosterone, known to govern libido and mating competence [17,18]. In ewes, the preovulatory surge of estradiol is a dependable indicator of behavioral estrus, a process tightly regulated by the hypothalamic–pituitary axis [19].

By synthesizing insights from both traditional and contemporary assessment methods, this review provides a comprehensive and practical perspective on fertility evaluation. It aims to equip practitioners and researchers with the knowledge necessary to inform effective decision-making in livestock management and the application of reproductive technologies [20,21]. We also explore approaches to address current methodological constraints and consider reproductive practices observed across diverse human populations.

## 2. Methodology

This comparative review employed a structured search strategy to identify relevant literature on fertility and semen assessment techniques, with a focus on cattle, buffalo, sheep, goats, and pigs. The primary aim was to identify, compare, and synthesize key methodological approaches and findings across these species. Searches were conducted in major scholarly databases (Elsevier/Scopus, Springer, Taylor & Francis, and MDPI) using combinations of key terms, including semen assessment, sperm quality, fertility, livestock, bull, ram, buck, boar, CASA, and sperm DNA integrity. The timeframe covered was 1938 to 2025. Inclusion was based on relevance to core assessment techniques (e.g., motility, morphology, chromatin integrity, and advanced assays) in the target livestock species. A total of 316 publications were selected for full-text analysis and form the basis of this synthesis. Table 1 categorizes the final corpus by source and dominant thematic focus.

## 3. The Concept of Fertility

Fertility extends beyond the biological capacity to reproduce (fecundity); it encompasses the successful birth of healthy offspring capable of surviving in challenging environments [22,23]. Fertility is fundamentally rooted in the biological processes of conception, gestation, and parturition, and is substantially influenced by both genetic and environmental factors [24,25].

A comprehensive understanding of fertility and reproduction across diverse species is essential for improving productivity, which depends primarily on pregnancy rates and the number of offspring born and successfully weaned [26]. As noted previously, knowledge of reproductive processes enables researchers, breeders, and producers to manage herds more efficiently and to produce animals with desirable traits and enhanced adaptability [27,28].

## 4. The Importance of Fertility

The rising consumer demand for meat can likely be addressed by enhancing fertility and reproductive efficiency within livestock herds. Reproductive efficiency constitutes a vital economic factor in livestock production. Sustaining optimal reproductive health within herds and flocks is therefore fundamental to the success of any production system [11,29]. Consequently, a thorough grasp of reproductive cycles and the function of sex hormones enables producers to enhance fertility and breeding efficiency, which in turn strengthens the broader animal production industry [30,31].

## 5. Spermatogenesis and Oogenesis

Gametogenesis is the process by which haploid reproductive cells or gametes are formed from diploid precursor cells within the gonads. This process, essential for sexual reproduction, occurs through meiosis and differs significantly between sexes [32,33,34].

**Figure 1:**
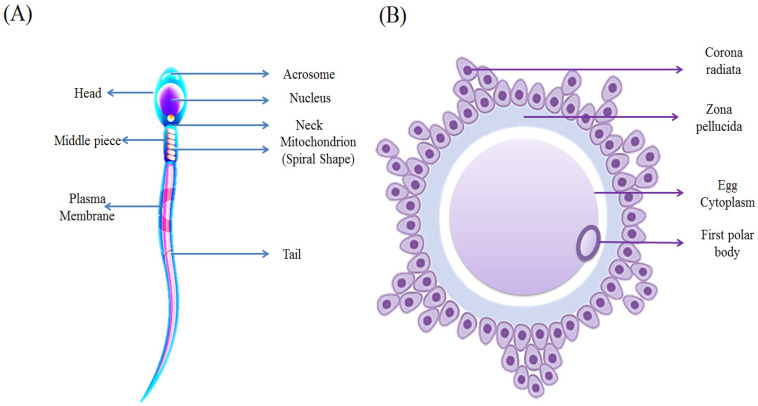
Morphology of the mature gametes. (**A**) The spermatozoon. (**B**) The ovum.

In males, spermatogenesis takes place in the testes, where germ cells (spermatogonia) undergo a series of divisions and transformations to produce mature spermatozoa (Figure 1A).

In females, oogenesis occurs in the ovaries. Here, germ cells (oogonia) develop into mature ova, or eggs (Figure 1B) [35]. A key difference between the processes is that oogenesis produces a single viable gamete per meiotic cycle, while spermatogenesis yields four.

The union of these two gametes, a spermatozoon and an ovum, during fertilization creates a diploid zygote. This cell is the progenitor of a new organism, dividing by mitosis and differentiating into all subsequent tissues [35]. A direct comparison of spermatogenesis and oogenesis is summarized in Supplementary File S1, Table S1.

## 6. The Fertility in Males

The male’s reproductive success depends on securing matings and achieving fertilization [36]. This success often varies widely between breeds and populations, primarily due to differences in the total number of ova fertilized over a lifetime [37]. In mammals, a common pattern is for a minority of males to sire most offspring each season [38,39].

There are two key factors that determine this outcome: (a) the number of accessible females and (b) the male’s fertilization capability [36]. Competition for mates drives the evolution of traits for male–male competition, such as increased size or weaponry [40]. It also selects for post-copulatory traits that improve sperm competitiveness, like higher sperm counts [41,42].

Research continues to investigate how selection shapes these morphological, physiological, and behavioral traits [40,43]. Additionally, considerable variation in fertility exists within breeds and populations, which directly impacts individual reproductive success [36].

Field studies have indicated that reduced fertility or transient infertility in males may be more prevalent in natural populations than previously acknowledged [44,45]. Male reproductive assessment typically includes measuring semen traits like volume, concentration, total sperm count, motility, viability, and morphology (including the proportion of normal sperm and those with a normal apical ridge; % NAR). Functional tests assess the acrosome reaction (% ARIC30 after 30-min ionophore stimulation), while kinematic parameters (curvilinear velocity/VCL, straight-line velocity/VSL, and average-path velocity/VAP) evaluate sperm movement. Relative testis size is also a common metric.

### 6.1. Morphological and Physiological Characteristics of the Male Reproductive System and Their Relation to Fertility

#### 6.1.1. Relationship Between the Size of Testicular and Fertility

Several studies reported that testicular size may be a good indicator of sperm production and thus fertility, which is consistent with observations on stallions, rams, and bulls. In this regard, we cannot underestimate the correlation between testicular size, body size, and genetic resources [46,47]. Abraham et al. [48] reported that testicular length has been used as an indicator (indirect indicator) of the onset of semen production.

#### 6.1.2. Physiological and Anatomical Differences

Also, there are the physiological and anatomical differences between different individuals, such as the prepuce that is adherent to the penis until two or three years of age, making protrusion of the penis unattainable in young male alpacas [49,50].

Seventy to eighty percent of the total mass of the testicle is in seminiferous tubules; thus, the testicular volume is reflected in spermatogenesis [51]. Several studies reported that there is a relationship between testicular volume and semen profiles in infertile human males; the measurements of testicular volume have been utilized as an indicator to estimate spermatogenesis [52,53].

#### 6.1.3. Measurement of Testicles

Nowadays, there are many methods for testes measurement, such as utilizing callipers (Supplementary File S1, Figure S1), a chidometer, or ultrasonography. On the other hand, there is the chidometry method as a traditional method [54,55]. As for the ultrasonographic method, several investigations confirmed that the calculation of testicular volume can be possible utilizing an ultrasonographic measurement formula, according to this equation: length ∗ width ∗ depth ∗ 0.71 (LWD0.71) [56].

### 6.2. Assessment of Sperm Characteristics

#### 6.2.1. The Technique of Collecting Semen

Standard techniques for semen collection include the electro-ejaculator (EE) and the artificial vagina (AV). Among these, the AV method is the most straightforward and widely employed. This technique is quick, painless, and avoids causing stress to the animal [33].

The artificial vagina resembles a car radiator hose and is approximately 6 inches in length. Inside the AV, there is an inner rubber liner that holds water heated to a temperature of 40–45 °C (113–113 °F), situated between the hose and the liner. This heated water serves to simulate the conditions of a female’s vagina. A latex rubber collection cone is positioned within the AV, and a graduated collection tube is attached to the end of the cone for gathering the semen [57,58] (Figure S2).

Maintaining semen samples at 37 °C is critical. Temperature deviations exceeding ±10 °C can significantly reduce sperm longevity and increase metabolic rates [59]. Exposure to temperatures above 50 °C causes irreversible loss of motility within minutes.

Recommended protocol for semen handling: (1) use a pre-warmed, dedicated collection tube for each male. (2) Minimize the time interval between sample collection and analysis. (3) Maintain sample temperature at approximately 37 °C using an insulated vial or warm water bath during transport. (4) Measure volume using the graduated collection tube [59,60]. The handling methods and initial evaluation for semen (manual semen assessment) are still the gold standard for new techniques. The following is a review of these tests in some detail.

#### 6.2.2. Progressive Sperm Motility

The new World Health Organization (WHO) manual from 2010 recommends categorizing sperm motility into three groups: non-progressive, progressively motile, and immotile, moving away from the previous grading system of (a), (b), (c), and (d). Technicians and researchers often find this new approach challenging for accurately assessing forward progression without bias [61].

Sperm motility is evaluated based on the following classifications: (1) immotility (IM): no movement observed. (2) Progressive motility (PR): sperm exhibiting active movement, either in a linear direction or in large circles, irrespective of speed [62]. (3) Non-progressive motility (NP): this category describes sperm that are moving but do not advance. Movement may include swimming in tight circles, subtle side-to-side head displacement due to flagellar beating, or general twitching without forward travel.

For a complete assessment, it is standard practice to evaluate total motility, which includes both PR and NP sperm [63,64].

##### Measuring Progressive Motility

Traditional sperm motility assessment [65] involves placing a semen drop on a slide pre-warmed to 38 °C, diluting with pre-warmed 2.9% sodium citrate solution, and covering with a pre-warmed 22 × 22 mm coverslip. Motility is scored subjectively by examining at least five fields under phase-contrast microscopy (200× or 400×) and recording the percentage of progressively motile sperm in 5% increments. Additional parameters quantified include average path velocity (VAP), curvilinear velocity (VCL), straight-line velocity (VSL), amplitude of lateral head displacement (ALH), beat cross frequency (BCF), percentage hyperactivation, linearity (LIN), and straightness (STR) [66].

#### 6.2.3. Sperm Concentration

Sperm concentration refers to the number of spermatozoa per unit volume of semen and represents a fundamental metric for assessing male fertility. This parameter varies considerably among species and serves as a key indicator of testicular function and reproductive potential [67].

Accurate measurement requires specialized equipment. The improved Neubauer hemocytometer, featuring dual counting chambers, is a standard tool for this precise quantification (Figure S3A,B) [68,69]. It is critical to differentiate between sperm concentration (sperm per milliliter) and total sperm number (sperm per entire ejaculate), as they represent distinct parameters (Table S2).

**Figure 2:**
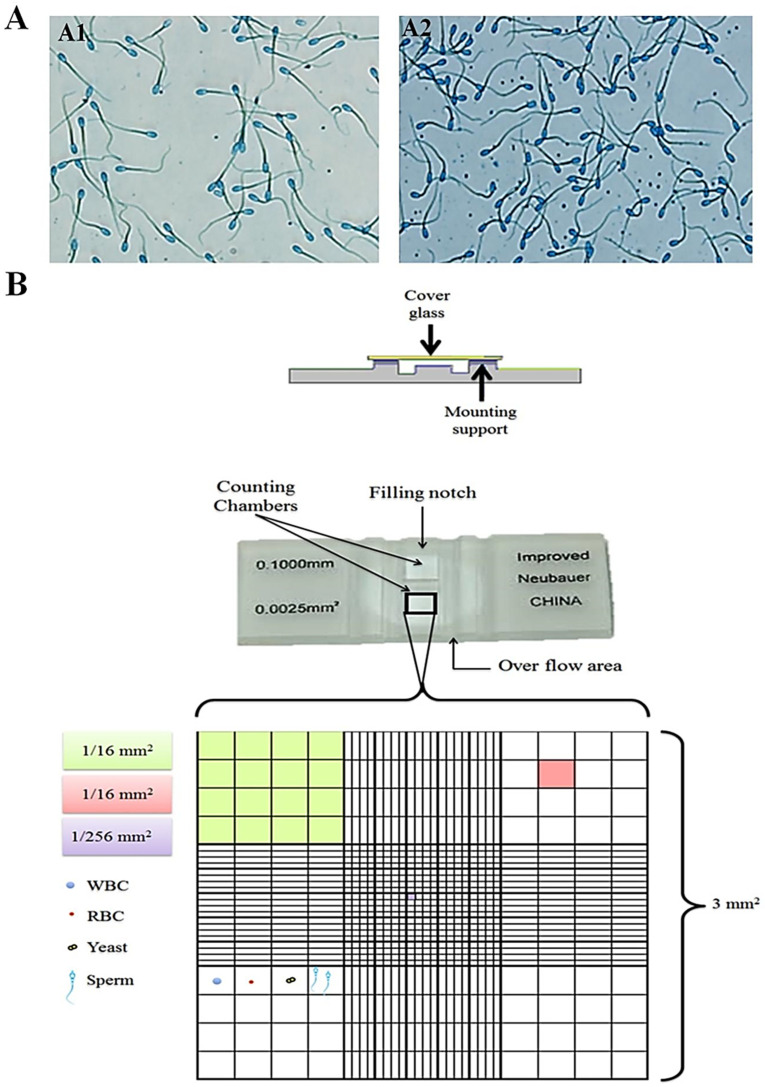
(**A**) Sperm concentration in (**A1**) Inner Mongolia Cashmere goats and (**A2**) Dazu black goats. (**B**) The improved Neubauer hemocytometer with different slide dimensions.

Breed-specific differences in this trait are well-documented. For instance, a comparative study of Inner Mongolia Cashmere and Dazu Black goats revealed clear variations in sperm concentration, assessed using an Olympus IX51 Inverted Microscope Fluorescence Phase Contrast 5MP (Olympus Corporation, Tokyo, Japan) with CellSens Standard software (version 4.4.) (Figure 2A) [68,69].

##### Methods for Defining the Concentration

Several methods are available for determining sperm concentration, each with varying complexity and accuracy (Table S2). The improved Neubauer hemocytometer remains a long-established, inexpensive reference method, though it is labor-intensive and requires skilled technique to minimize counting errors. Modern dedicated systems, such as the NucleoCounter^®^, utilize fluorescence-based detection of DNA stains to provide rapid, highly reproducible, and user-independent counts, making them valuable for high-throughput laboratories. For routine analysis, purpose-built chambers such as the Makler^®^ or disposable counting slides (for example, Leja^®^) offer standardized depth and grid patterns that simplify the process and improve consistency compared to traditional hemocytometers. While spectrophotometric (photometric) methods are fast, they require species-specific calibration and can be less accurate at extreme concentrations [68,69]. The choice of method depends on the required precision, sample throughput, available resources, and the need for compliance with specific industry or breeding program standards.

The hemocytometer is regarded as an excellent standard for assessing spermatozoa counts. Following the dilution of the sample in a suitable clear diluent, the hemocytometer is employed to ascertain the concentration of spermatozoa in an ejaculate. The hemocytometer features red blood cell (RBC) counting chambers, with each primary counting area containing 25 subdivisions arranged in a 5 × 5 grid pattern. These subdivisions are further partitioned into 16 smaller counting units organized in a 4 × 4 configuration. This hierarchical structure results in 400 individual counting units within each primary chamber (calculated as 25 × 16), creating a systematic grid for accurate cell enumeration, as demonstrated in Figure 2B.

The total area of the four hundred tertiary squares on the hemocytometer corresponds to 1 mm^2^. When a drop is placed under the coverslip in the counting chambers (Neubauer cell), the thickness of the fluid film in this chamber is 0.1 mm. Therefore, the total volume of semen covering the four hundred tertiary squares in the red blood cell (RBC) chamber is 0.1 mm^3^.

The hemocytometer remains a common method for determining sperm concentration. The general principle involves diluting a semen sample in a fixative or immobilizing solution, loading it into a chamber of known depth and grid area, and manually counting sperm within defined grids under a microscope. The concentration is then calculated using standard formulas that account for the dilution and chamber volume. It is important to note that specific protocols for diluent composition, dilution factor, and the number of grids counted can vary between laboratories and species-specific guidelines [67,68,69,70,71].

Some equations:

1- *n* = Number of spermatozoa cells counted in five secondary squares.
2- *n* × 5 = Number of spermatozoa cells in (twenty-five) secondary squares.
3- *n* × 5 × 200 = Number of spermatozoa cells in 0.1 mm^3^ of undiluted sample.
4- *n* × 5 × 200 × 1000 = n × 10 × 1,000,000 = Number of spermatozoa cells in 1 cc of undiluted semen.

#### 6.2.4. Viability (Live/Dead)

Sperm membrane integrity is an indicator of the viability of samples using a test of dye-exclusion. This is necessary because less than about forty percent of sperm are progressively motile. It is vital to know whether immotile sperm are dead or alive [72].

##### Viability (Live/Dead) Assessment

**Figure 3:**
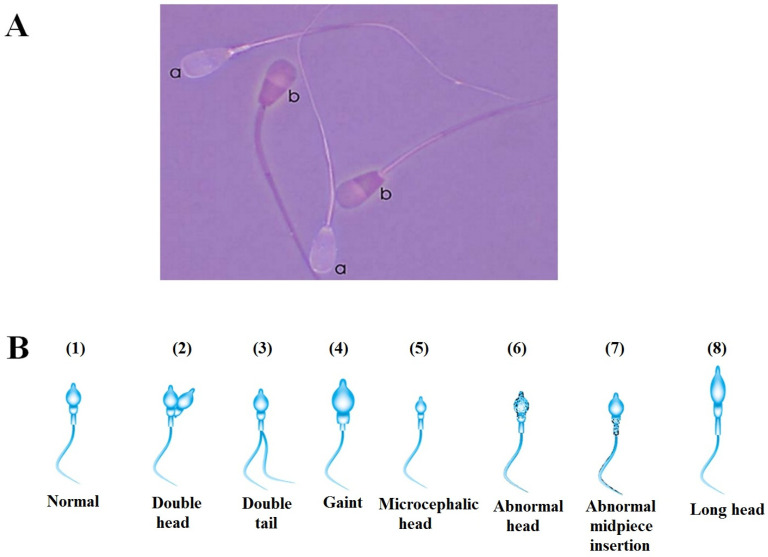
(**A**) Semen smear stained with eosin-nigrosin for evaluation of the acrosome membrane demonstrating sperm with the following: (a) intact acrosome (Live) and (b) lost acrosomes ‘react acrosomes’ (dead). (**B**) Schematic representation of common sperm morphological abnormalities: (1) normal, (2) double head, (3) double tail, (4) giant, (5) microcephalic head, (6) abnormal head, (7) abnormal midpiece insertion, and (8) long head.

Sperm viability is assessed using a live/dead staining technique [73,74]. In this method, a semen sample is mixed with eosin-nigrosine stain on a warmed slide and smeared. After drying, sperm are examined under a light microscope. Viable (live) sperm remain unstained, while non-viable (dead) sperm absorb the pink eosin dye (Figure 3A) [75,76].

Eosin-Nigrosine Solution Preparation: The staining solution is prepared by dissolving sodium chloride (NaCl) and eosin Y in distilled water, adding nigrosine, boiling, then filtering, and storing in a dark bottle [77,78] (Table S3).

Procedure: Pre-warmed semen is mixed with the stain and incubated briefly. A droplet is smeared on a slide, air-dried, and approximately 200 sperm are counted. Pink sperm are classified as dead, clear sperm as live. Sperm with only a localized pink neck region are considered non-eosinophilic [77,78,79].

#### 6.2.5. Sperm Morphology (Normal/Abnormal)

Sperm morphology is evaluated against standardized criteria (e.g., WHO) to classify normal and abnormal forms, with the proportion of normal sperm linked to fertilization success [80,81].

A normal spermatozoon is defined by the following characteristics [80,81,82,83]: (a) head: smooth, oval contour, 4–5 µm in length, and ~2.5 µm in width (length-to-width ratio of 1.5–1.75). A well-defined acrosome covers 40–70% of the head. (b) Mid-piece: slender, measuring less than 1 µm in width. (c) Tail: a uniform, straight, uncoiled tail approximately 45 µm long, thinner than the mid-piece.

Head abnormalities encompass double heads, duplicate bodies, giant or micro sperm, rough head surfaces, abnormal mid-pieces, elongated heads, and vacuolated heads (vacuoles occupying less than 20% of head area), plus heads with reduced acrosomal areas (exceeding 40% of head area), or combinations of these defects.

Mid-piece and neck abnormalities include bent neck configurations (tail and neck forming angles exceeding 90° to the sperm body axis), thick or irregular mid-pieces, and asymmetrical mid-piece insertion into the head lacking mitochondrial sheaths.

Tail abnormalities comprise hairpin formations, shortened or fractured tails, multiple tails, bent tails (90° angles), twisted configurations, or tails with irregular width variations [75,76,84] (Figure 3B).

##### Sperm Morphology Test

The same slide used for the eosin-nigrosine stain can be employed to screen for morphological abnormalities in spermatozoa. A drop of immersion oil is placed on the coverslip, and the semen sample is examined under bright-field microscopy at 100× magnification.

Proper slide preparation requires achieving appropriate thickness; excessively thick preparations hinder accurate assessment, as many spermatozoa may rest on their edges. Ideally, samples should be flattened to permit optimal visualization and evaluation.

Since individual spermatozoa may not lie perfectly within one focal plane, the microscope’s fine focus must be adjusted for each cell during assessment. Sperm morphology is assessed by classifying individual cells as normal or abnormal based on strict criteria for head, midpiece, and tail structure [80,81,82,83]. A key aspect of this assessment is the sample size, with common standards recommending the evaluation of either 100 or 200 spermatozoa from multiple microscope fields to calculate the percentage of normal forms. This variation in the recommended number of cells to count exemplifies the lack of a single, universal technical standard, highlighting the need for consistency within a given laboratory or breeding program [85,86].

#### 6.2.6. Capacitation Status

To achieve fertilizing capacity, mammalian spermatozoa must complete a final maturation process known as capacitation. This occurs naturally within the female reproductive tract and involves essential biochemical and physiological modifications [87,88]. Key changes include the regulation of intracellular ions, increased fluidity of the plasma membrane, a transition to a hyperactivated pattern of motility, and shifts in metabolic pathways [89].

Only capacitated sperm can effectively bind to the zona pellucida of an oocyte and undergo the subsequent acrosome reaction, which is required for penetration and fertilization.

The molecular mechanism of capacitation is coordinated by several interdependent signaling pathways. Principal regulatory factors, such as modulations in intracellular pH [89], influxes of calcium ions (Ca^2+^) [90], the removal of cholesterol from the sperm membrane [91], activation of cyclic AMP (cAMP) pathways [92], dynamics of actin polymerization [93], and increased protein tyrosine phosphorylation [94,95].

##### Hypo-Osmotic Swelling (HOS)

A functional plasma membrane is vital for sperm fertilization capability. This function can be assessed by testing the membrane’s permeability to water using the hypo-osmotic swelling (HOS) assay [96]. The test evaluates a sperm cell’s ability to maintain integrity under moderate hypo-osmotic stress [97].

When placed in a hypotonic solution, viable spermatozoa with intact membranes absorb water, leading to characteristic swelling and coiling of their tails. Non-viable spermatozoa with compromised membranes show no such reaction [98]. This makes the HOS test a valuable supplemental measure of sperm viability, especially in cases of severe asthenozoospermia or immotile cilia syndrome, where motile sperm are scarce or absent [99].

##### Hypo-Osmotic Swelling Test

The test is performed by mixing 0.1 mL of semen with 1 mL of a hypo-osmotic solution (150 mOsm kg^−1^; Table S4) and incubating the mixture at 37 °C for one hour. Following incubation, a drop of the solution is placed on a slide under a coverslip for examination. Spermatozoa with functional membranes will exhibit characteristic tail curling or swelling due to water influx. Approximately 200 spermatozoa are assessed across multiple fields at 400× magnification. The percentage of reacted (swollen) spermatozoa is calculated using the following formula [100,101]:

$$The\;swollen\;spermatozoa=\frac{Number\;of\;reacted\;cells}{The\;total\;spermatozoa\;counted\;in\;the\;same\;area}\times 100$$

#### 6.2.7. Acrosomal Integrity

Acrosomal integrity can be assessed through two complementary approaches using eosin-nigrosin-stained semen smears. (a) Dual-Purpose Staining: Thin semen smears prepared for standard sperm viability evaluation (eosin-nigrosin stain) can simultaneously serve to assess acrosome integrity. Acrosomes are categorized as follows based on established morphological criteria: (1) normal (live): acrosomes appearing smooth and fully intact across the sperm head. (2) Reacted/lost (dead): acrosomes exhibiting loss with a distinct “shoulder” visible at the equatorial segment region [102] (Figure 3A). (b) Staining is carried out according to [103] (Table S5). The preparation of stain: Giemsa stain with absolute methanol in a pestle and mortar, then adding glycerol. Then, this mixture is incubated at 37 C for 7 days, thus shaking this mixture for two minutes each day.

#### 6.2.8. Sperm Plasma Membrane Integrity

One key characteristic that distinguishes live cells from dead ones is the loss of motility and the physical integrity of their plasma membranes; dead cells exhibit these deficiencies [104,105,106]. The integrity and proper functioning of the outer spermatozoa plasma membrane are essential for several physiological processes, including sperm metabolism, binding to the ova, the acrosome reaction, and capacitation [107,108]. Therefore, the assessment of plasma membrane integrity, in conjunction with sperm morphology traits, can be instrumental in predicting the fertilizing ability of spermatozoa.

#### 6.2.9. Sperm Migration

##### Processes Inside the Testis

Spermatozoa are produced by spermatogonia in the testis, which undergo meiotic and mitotic divisions within the seminiferous tubules. Spermatogenesis can be divided into two successive stages. (1) Spermatocytogenesis: this stage encompasses the development of cells from spermatogonia to the formation of secondary spermatocytes [109]. (2) Spermiohistogenesis/spermiogenesis: this phase involves the maturation and differentiation of spermatozoa, beginning with the spermatid stage [110].

Regarding the temporal progression of spermiohistogenesis, the approximate sixty-four-day cycle can be subdivided into four distinct phases. (a) Mitosis of spermatogonia: this phase lasts for sixteen days, culminating in the formation of primary spermatocytes [111]. (b) First meiosis: this phase extends for twenty-four days and involves the division of primary spermatocytes into secondary spermatocytes. (c) Second meiosis: this phase occurs over a few hours and results in the generation of spermatids. (d) Spermiogenesis: this final phase lasts for twenty-four days, culminating in the formation of fully developed sperm cells [112].

##### Spermatocytogenesis

**Figure 4:**
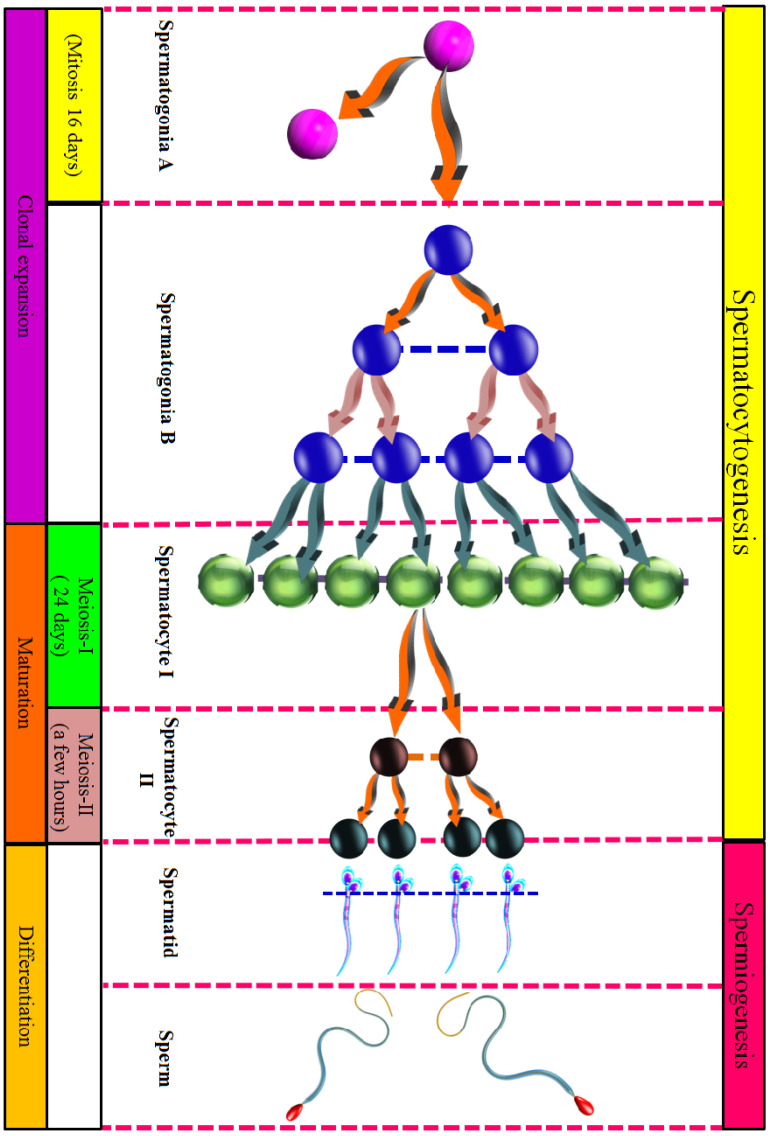
The stem cells of the germinal cells lie on the basal lamina of the seminiferous tubules (convoluted tubules), under the name of type A spermatogonia. Those cells undergo mitosis: one of those daughter cells renew the stock of spermatogonia (type A), the other becomes spermatogonia (Type B). These divide, and the daughter cells migrate towards the lumen. In roughly sixty-four days, they differentiate themselves into sperm cells up to the outer surface of the epithelium.

Among the over one billion spermatogonia present in the testes, these cells form the basal layer of the germinal epithelium [113]. Several types of cells can be distinguished: (1) type A spermatogonia: these cells undergo mitotic division to self-replicate through a process known as homonymous division, preserving the population of spermatogonia. The initiation of spermatogenesis occurs through a heteronymous division, in which daughter cells (type A cells of the second group) remain connected through cytoplasmic bridges [113]. This cytoplasmic connectivity is essential for the induction of spermatogenesis. (2) Type B spermatogonia, generated mitotically from type A spermatogonia, themselves divide mitotically to form primary spermatocytes (I). These primary spermatocytes immediately commence meiosis. During the initial S phase (preleptotene), key events include DNA duplication and their migration from the basal compartment towards the lumen. Subsequently, they enter the extended prophase I of meiosis, reaching a stage where they are observable using a light microscope [114,115], (Figure 4).

The testes are composed primarily of seminiferous tubules, the site of spermatogenesis, and interstitial tissue containing Leydig cells for testosterone production. The total mass and volume of the tubular compartment are functionally significant as they correlate with spermatogenic output, which is why testicular size/scrotal circumference is a common, practical field indicator of sperm production potential in bulls, rams, and bucks [46,47,51,116].

##### Processes in the Epididymis

After sperm production in the seminiferous tubules, spermatozoa are collected and stored in the ductus epididymidis. Several maturation steps occur in the epididymis, including the following: (1) the genetic material (DNA) becomes more condensed due to protein deposition in the nucleus [117]. (2) The heads of the sperm cells decrease in size and become more compact, which is vital for the proper decondensation of paternal DNA in the oocyte during fertilization. (3) The residual cytoplasm is reduced, leading to a more-slender sperm morphology. (4) Sperm motility is initiated, although it is inhibited by the surrounding environment. (5) The structure of the plasma membrane undergoes changes [116,118], which enhances the sperm’s motility, acrosome reaction capability, and capacitation [119].

##### The Ejaculation Path

The ejaculation pathway for sperm consists of two vas deferens, which converge into the ejaculatory duct and then into the urethra. The ejaculatory duct serves as the canal that passes through the prostate gland and connects to the urethra. Several glands along this path add their fluids to the semen. These include the seminal vesicles, prostate gland, Cowper’s glands, and Littre’s glands [120,121,122].

##### Through the Utero-Tubal Junction (UTJ)

Mammalian sperm undertake a significant journey from the uterine cavity to the fertilization site in the oviduct. To become fertilization-competent, sperm require a period of residence within the female tract, undergoing a unique mammalian process called capacitation [123,124]. Studies show that removing glycophosphatidylinositol (GPI) proteins (GPI-APs) triggers this capacitation [125]. Within the immunologically active female reproductive tract, sperm are shielded by surface glycoproteins like CD59, CD52, and CD55. These protective molecules originate from epididymal fluids [126]. After traversing the uterotubal junction (UTJ), sperm navigate the oviduct, moving through the isthmus towards the ampulla.

For sperm to successfully traverse the oviduct and reach the oocyte, they must exhibit adequate motility [127,128]. This journey is challenging, as sperm must swim against the current generated by cilia lining the oviductal walls.

Sperm–oviduct interactions have been extensively investigated. Kirchhoff et al. [126] observed that mouse sperm do not traverse the oviduct continuously; rather, they intermittently adhere to and release from the isthmus lining, potentially multiple times during transit. This observation suggests that hyperactivation of the transition to powerful, erratic swimming may facilitate detachment from the oviductal epithelium.

Meanwhile, other studies using sperm tagged with fluorescent dye (so scientists could track them) showed something else. Refs. [129,130] observed sperm being pushed back and forth within the isthmus. This back-and-forth movement happened along with rhythmic squeezing (peristaltic contractions) of the oviduct muscle itself. This tells us these muscular contractions play a really important role in helping sperm move along their path. Conversely, Ref. [130] found sperm located in the ampulla of the oviduct even when peristaltic movement was inhibited by the anticholinergic drug Padrin, highlighting that spermatozoa possess the inherent capability to migrate through the oviduct independently.

Several studies reported that there are many genes related to sperm migration through the uterotubal junction (UTJ), such as *Tpst2* [131], *Tex101* [132], *Rnase10* [133], *Prss37* [134], *Pmis2* [135], *Pgap1* [136], *Pdilt* [137], *Ly6k* [138], *Clgn* [139], *Calr3* [140], *Adam1α* [141], *Adam2* [142], *Adam 3* [143], and *Ace-t* genes [144]. These factors are crucial for sperm migration through the uterotubal junction (UTJ).

##### Migration-Sedimentation Test (MST)

**Figure 5:**
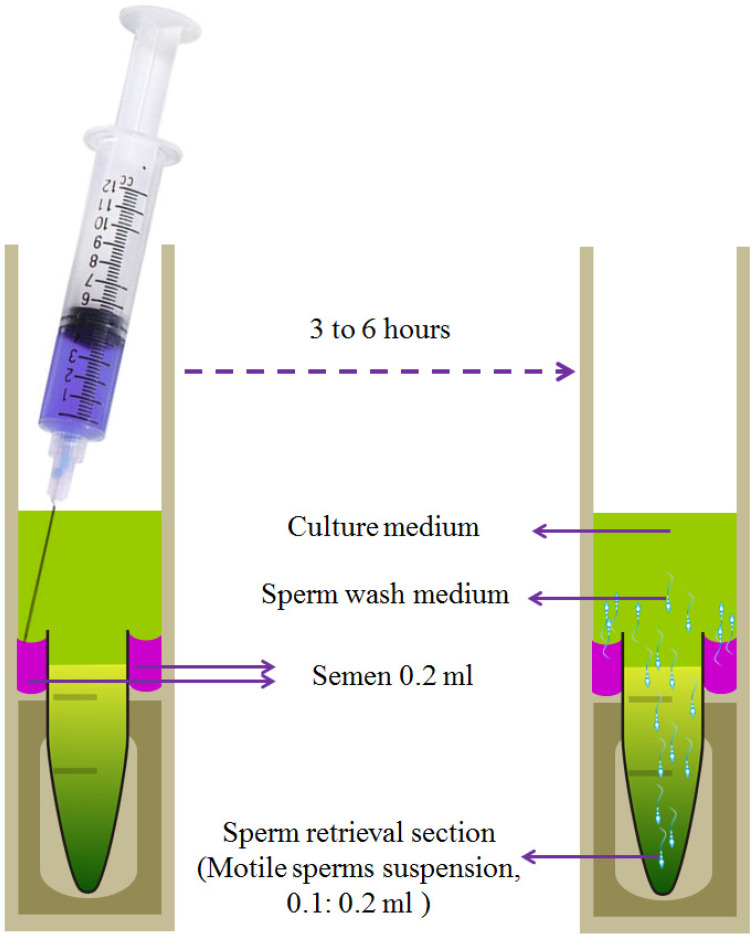
Drawing of a migrationsedimentation (MS) tube and its chambers for loading.

A method for collecting motile sperm, adapted from the design by Tea et al. [145], is illustrated in Figure 5. The procedure is as follows: (a) prepare two Pyrex glass tubes filled with B2 culture medium. (b) Load approximately 1 mL of semen into a sterile syringe. (c) Carefully layer the semen sample at the bottom of the outer tube, ensuring that the top of the semen layer is positioned about 1 mm below the rim of the inner tube.

Incubation is then conducted at 37 °C for 30 min, followed by a completion period at room temperature for three to six hours. Sperm suspension can subsequently be collected by aspiration from the conical tube.

This technique, referred to as migration-sedimentation (MS), is employed to separate motile spermatozoa without the need for centrifugation. It primarily relies on the motility of sperm and their migratory behavior. Where, separated sperm are usually characterized by their motility, concentration (106 mL^−1^/human samples), normal morphology, and degree of motility from (1 = poor: 4 = excellent) [146].

#### 6.2.10. Chromatin Integrity

The standard of analysis evaluates sperm concentration, vitality, morphology, and their motility. One of the most vital parameters of sperm in their fertilizing potential is chromatin integrity, which has an indirect or direct positive correlation with fertility, including fertilization rate, pregnancy, embryo quality, and successful delivery rate. Where DNA chromatin integrity for spermatozoa provides more predictive, prognostic, and diagnostic approaches than standard semen analyses. Understanding the chromatin structure, etiology, abnormality, identification factors that disturb chromatin integrity for sperm, and the mechanism of their action may help in recognizing the cause of infertility [147].

##### Assessment of Chromatin Integrity

Chromatin integrity is typically evaluated using Chromomycin A3 (CMA3) as follows [148]. (a) Fixation: Prepare air-dried sperm smears. Soak slides in methanol-acetic acid (3:1) for 5 min. Remove excess fixative. (b) Staining: Treat smears with CMA3 solution (0.25 mg/mL in McIlvaine buffer, pH 7.0, 10 mM MgCl_2_). Air-dry. (c) Mounting: Apply mounting medium to the smear and cover with a coverslip. (d) Analysis: Assess the slides using fluorescence microscopy. Evaluate the chromatin status in approximately 400 spermatozoa.

Spermatozoa with abnormal chromatin integrity will show positive CMA3 fluorescence over the head. Normal spermatozoa, with intact chromatin, will display negative or only faint, nonspecific fluorescence [149,150,151].

#### 6.2.11. DNA Integrity

Nuclear abnormalities in spermatozoa significantly impair reproductive outcomes, encompassing structural/numerical chromosomal aberrations, DNA strand breaks, and Y-chromosome microdeletions. The assessment of spermatozoal DNA damage has emerged as a critical indicator of semen quality. Primary forms of DNA damage include the following: (1) double- and single-strand DNA fragmentation, (2) oxidative modifications (e.g., alkylation), and (3) DNA–protein crosslinks [152,153]. Multiple methodologies evaluate sperm DNA integrity: (1) terminal deoxynucleotidyl transferase dUTP nick end labeling (TUNEL) [154], (2) the sperm chromatin structure assay (SCSA) [155], (3) sperm chromatin dispersion (SCD) test [156], and (4) DNA breakage detection [157].

Techniques such as the neutral comet assay, TUNEL, and denaturation-based tests (SCSA, alkaline comet, and SCD) are specifically designed to quantify the extent of DNA damage [158]. An indirect assessment of DNA integrity can also be made by examining morphologically normal, motile sperm, either through separate analysis or simultaneous DNA-morphology evaluation [159,160].

#### 6.2.12. Seminal Plasma and Sperm Membrane Proteins

Seminal plasma contains a diverse array of organic and inorganic components, with high-molecular-weight proteins serving critical functional roles. The specific composition of these proteins varies across species. They are essential for key sperm functions, including the acquisition of fertilization competence (capacitation), the regulation of motility, tolerance to cryopreservation, the maintenance of viability, and overall sperm protection [161,162,163,164].

The importance of these proteins is supported by observed correlations between their concentrations in seminal plasma and standard semen quality parameters [165,166]. Proteins on the sperm membrane itself are equally crucial. They facilitate the recognition and binding to the zona pellucida and oolemma and are involved in inducing the acrosome reaction [167]. Worth mentioning, damage to the sperm plasma membrane, which is often detectable through protein dysfunction, is strongly associated with idiopathic male infertility, even in cases where routine semen analysis appears normal [168].

## 7. Outcomes of Laboratory Semen

Most assays of spermatozoa show moderate correlations with fertility traits. The relationships between various laboratory assay results and the fertility or infertility of semen, particularly those that have been cryopreserved, have been established since the 1950s [169,170]. These correlations have proven statistically significant, revealing considerable variability; for instance, the correlation between fertility and sperm motility has been reported to range from 0.15 to 0.83 [171,172].

Studies utilizing computer-assisted semen analysis (CASA) on standardized samples from AI bulls indicate that correlations between individual post-thaw motility patterns and fertility measures (e.g., field fertility and linearity) are variable, with reported r^2^ values ranging from 0.45 to 0.63 [173,174]. However, combining assessments of multiple motility patterns significantly strengthened these correlations, yielding r^2^ values between 0.68 and 0.98 [175]. Predictive capacity further improved, reaching an r^2^ of 0.83, when motility data were integrated with other functional parameters of spermatozoa [174].

In contrast, correlations between fertility and sperm morphology have exhibited considerable variation as well (0.06 to 0.86) [176]. Conversely, the quality grade of semen tends to yield statistically non-significant results in most cases [177,178,179,180,181].

### Evolution of Assessment Paradigms: From Foundational Morphology to Functional and Molecular Assays

The assessment of semen and fertility has evolved through distinct phases, moving from gross morphological evaluation to dynamic functional and molecular analysis. The foundational period (mid-20th century) established the core manual techniques that remain reference standards today: visual motility estimation, concentration determination via hemocytometer, and morphology assessment using stains such as eosin-nigrosin [59,65]. The late 20th century introduced the first major technological shift with the development of CASA, which provided objective, kinematic data on sperm movement, moving beyond subjective scoring [182,183]. Concurrently, functional integrity tests, such as the HOS test and capacitation assays, gained prominence [96,100]. The contemporary, 21st-century paradigm is characterized by molecular and omics approaches, including assays for sperm chromatin integrity (SCSA and TUNEL), DNA damage, and seminal plasma proteomics, which seek to identify biomarkers of fertility beyond what traditional microscopy can reveal [155,158,163].

## 8. New Fertility Techniques for Measuring

On the other hand, profitability and productivity are assessed through metrics such as the conception rate, ovulation rate, and number of offspring born. The survival of species depends on reproduction, a multifaceted biological process. This process encompasses several critical phases: the synthesis of sex hormones such as testosterone and estrogen, the development of reproductive tracts, gametogenesis, fertilization, gestation, and parturition [11,22].

Given the economic value of animal production, a major research focus has been to define measurable semen characteristics that correlate with male fertility in livestock [179]. This pursuit has led to significant research focused on understanding how characteristics of the ejaculate and semen can predict conception success or diagnose reproductive failures. Studies consistently show that key factors like sperm concentration, semen volume, sperm motility, acrosomal status, and sperm morphology play crucial roles in determining male fertility potential [180,181]. Beyond these fundamental assessments, a suite of advanced techniques now exists for detailed semen evaluation. Among the most sophisticated.

### 8.1. Computer-Assisted Semen Analysis (CASA) Systems

CASA systems combine specialized software with hardware, typically a microscope and high-resolution camera, to analyze semen samples [182]. A primary strength of CASA is its ability to precisely track sperm motion velocities and quantify overall motility patterns. This is an approach that refers to a variety of automatic analyses, which indicates fertility in males [183]. CASA has been applied to examine sperm in fish water samples [184], support animal breeding programs [185,186], and improve human reproductive technology [187]. It is also frequently used for pre-artificial insemination (AI) analysis [185,186,188]. CASA outputs include linear velocity, percentage of motile sperm, head frequency, and curvilinear velocity, derived from motile sperm trajectories [183]. This technique requires a costly and sensitive device, which is not available in most far research areas with limited settings or rural areas.

#### 8.1.1. CASA Application Based on a Smartphone (iSperm)

New smartphone-dependent technologies may overcome many problems, thus, the development of the systems of sperm motility analysis [183]. For example, CASA applications on smartphones are currently available commercially, such as iSperm, which works on Apple iOS “8” (Figure S4). The iSperm application requires just a smartphone with a high lens using this system, then we can evaluate the percentage of sperm motility, but we cannot know about the sperm trajectory. Using this system, sperm motility can be analyzed in far research areas with limited settings and rural areas. Analyses utilizing the CASA application are considered a good indicator for fertility [189,190]. Although the problem with the iSperm application is its inability to discover the sperm trajectory; however, it is of low cost (USD 1000) compared to CASA, which is approximately USD 10,000 (in 2017).

#### 8.1.2. CASA vs. Manual Methods/The Difficulties in the Seminological Assessment for Semen

Foundational manual semen assessment, while historically the gold standard and still widely used, is prone to subjective error and variability [187,191,192,193]. The evolution towards automated, objective analysis is exemplified by CASA systems, which were developed to address these limitations. CASA provides reproducible kinematic data, though it often validates its parameters against the foundational benchmarks established by manual techniques [194].

### 8.2. MTT [3-(4,5-Dimethyl-thiazol-2-yl)-2,5-diphenyl-tetrazolium-bromide) Assay]

The MTT assay is a colorimetric technique used to measure cell metabolic activity and cell proliferation [195]. Under specific conditions, NAD-(P)-H-dependent cellular oxidoreductase enzymes provide an indication of the total number of viable cells present. Tetrazolium dye assays, like the MTT assay, rely on specific enzymes within living cells to drive the conversion of the yellow MTT dye into an insoluble purple formazan compound [196] (Figure S5). Beyond measuring cell viability, these assays are valuable tools for gauging the effects of toxins, potential drugs, and cytotoxic agents [197].

The MTT assay must be performed under low-light conditions due to the reagent’s photosensitivity. In viable cells, the yellow MTT tetrazolium is reduced to purple formazan crystals [198]. These crystals are then dissolved using a solubilization solution such as SDS in acid, acidified ethanol, or DMSO to create a colored liquid. The resulting color intensity, measured spectrophotometrically at 570–600 nm, corresponds to the number of living cells. Solvent choice can affect absorbance readings [199]. A modified MTT assay has also been successfully used to assess sperm viability. In one study, this method was compared with the hypo-osmotic swelling test and eosin-nigrosin staining [200].

### 8.3. Semen Evaluation

Semen evaluation is an integral part of routine reproductive soundness assessments, used to diagnose fertility issues and facilitate AI. The primary objective of semen analysis is to identify one or more parameters that can reliably predict the fertilizing ability of the semen. Over the past few decades, numerous methods have been examined, but only a select few have been adopted for practical applications. Most investigations have focused on light microscopic evaluations of classical semen parameters, including motility, sperm morphology, concentration, and viability. Ref. [201] proposed a model comprising two key parameters: quantitative semen characteristics and qualitative semen traits.

There are several measures that can be considered to judge the fertility of females in animals, such as litter size (twining rate), fertility, kid mortality rate, and days open, can be calculated from the following equations [202,203,204] (Table S6).

## 9. Physiological Factors of the Female Reproductive System

The female reproductive tract in the primary livestock species covered (cattle, sheep, goats, and buffalo) is characterized by a bicornuate uterus. This Y-shaped organ is critical for pregnancy and features uterine horns, a short body, and a cervix. A key anatomical feature in ruminants is the presence of uterine caruncles, aglandular raised areas that form the attachment sites for the fetal placenta, and intercaruncular endometrium, which contains the glands that secrete histotroph essential for early conceptus development [205,206,207,208,209]. This specialized architecture underpins the synepitheliochorial placentation characteristic of these species. In contrast, pigs have a longer, more tortuous bicornuate uterus without discrete caruncles, reflecting a diffuse epitheliochorial placentation [210,211,212,213,214]. Uterine development and maturation, culminating in the functional endometrium, myometrium, and perimetrium, are well-documented in livestock models [215,216,217,218,219,220].

## 10. Histological, Histopathological, and Endocrine-Factors of the Female Reproductive-System

The female reproductive system (FRS) is complex and relies on a diverse array of hormones that influence the tissues involved in female reproduction, which can present interpretative challenges [221].

Endometrial provision of metabolic substrates, including glycogen, is critical for embryo survival and development across mammalian species, serving as an energy source prior to implantation [222]. In livestock, inadequate uterine endometrial function and secretory capacity, including nutrient provision, represent a primary cause of early embryonic mortality, resulting in failed pregnancy or subfertility [208,210,211]. Histopathological assessments, analogous in principle to human diagnostic approaches [223,224], are less common in production settings; instead, female fertility is typically evaluated through integrated reproductive performance metrics such as conception rate and days open.

### 10.1. Assessment of Female Fertility in Livestock

In contrast to males, female fertility is often assessed through performance metrics rather than direct gamete analysis. Key measures include conception rate, calving/lambing/kidding/farrowing rate, days open (the interval from parturition to subsequent conception), and litter size in multiparous species [202,203,204]. These metrics integrate the success of ovulation, fertilization, uterine receptivity, and early embryonic development.

### 10.2. Endocrine and Pathological Factors

Endocrine function is central to female fertility [225,226,227,228]. Disorders such as the development of cystic ovarian follicles or persistent corpus luteum are major causes of infertility in dairy cattle, disrupting normal estrous cyclicity [226,227]. Uterine health is equally critical; postpartum infections like metritis or endometritis directly impair fertility by creating a hostile environment for the embryo. Furthermore, adequate endometrial gland development and secretory function are essential for histotroph production and conceptus survival, particularly during early pregnancy in ruminants [208,210]. Table S7 summarizes key reproductive traits and common fertility parameters across the featured livestock species.

## 11. Artificial Insemination (AI)

### 11.1. The Definition of Artificial Insemination (AI)

**Figure 6:**
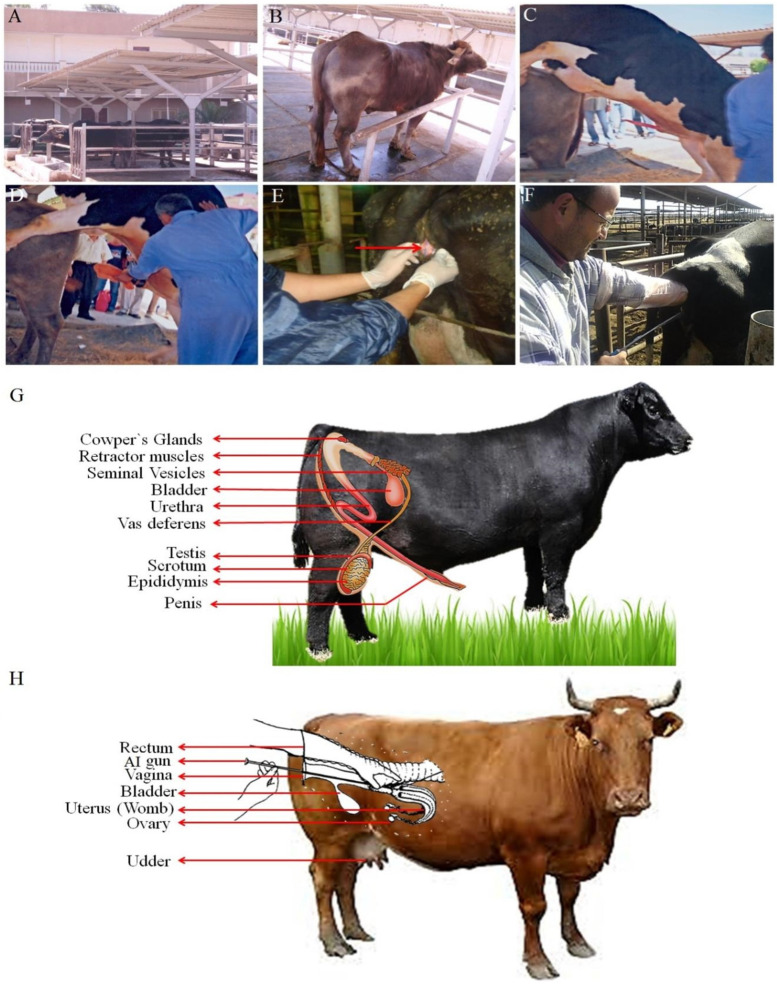
Steps of artificial insemination in cattle and buffalo: (**A**) select and prepare the male that has target traits, (**B**) bring the female to collect semen, (**C**) allow the male to jump to collect semen, and (**D**) collect the sample of semen utilizing an artificial vagina (A.V) for cattle, which is indicated in Figure S2B. (**E**) Controlled internal drug release (CIDR) technology is employed alongside hormonal treatments to synchronize estrus in females, effectively addressing various breeding challenges. The red arrow indicates the CIDR device, a progesterone-releasing vaginal insert. This T-shaped silicone rubber device is used to synchronize estrus (heat) cycles in cows and heifers by delivering controlled doses of progesterone. This includes difficulties with inefficient heat detection, poor responses to breeding programs in cows, females that are at incorrect stages of their estrus cycles when breeding commences, and females that are not cycling at all. Additionally, it is used to facilitate fixed-time AI, (**G**) the male reproductive tract system, and (**F**,**H**) artificial insemination in cows.

AI is the breeding of a sample of semen that has been collected from a special male with a female and inseminated into the vagina or uterus utilizing equipment or instruments (Figure 6) rather than by natural way (breeding) [229].

The primary rationale for employing AI is to enhance gamete density at the site of fertilization [230,231]. Over several centuries, numerous pioneers have significantly contributed to the scientific development of AI for both humans and livestock [232]. The progression leading to the widespread use of AI today is rooted in experimental research conducted many years ago [233]. It is important to note that many modern techniques used in human AI programs have been adapted from studies on various livestock species, as farmers aimed to enhance animal production, particularly in milk output, through the use of AI with semen from selectively bred males exhibiting desirable genetic traits [234].

### 11.2. Advantages and Disadvantages of Artificial Insemination

AI is now widely utilized in animal production due to its practical benefits. Several studies confirm many advantages, such as better disease management, enhanced safety for handlers and animals and the resolution of breeding difficulties in stallions through semen collection and AI [235,236].

Conversely, there are several drawbacks associated with this method, such as the necessity for specialized equipment and technical expertise. Furthermore, improperly executed AI can lead to various complications [237,238].

## 12. Integration of Advanced Diagnostic Techniques in Male Fertility Assessment

The comprehensive evaluation of male fertility potential in livestock has evolved beyond foundational semen analysis to include a suite of advanced functional, molecular, and genomic assays. This integration is crucial for accurate prognosis, informed breeding decisions, and unraveling the complex etiology of subfertility [237,238,239,240,241].

### 12.1. Advanced Methodologies for Core Semen Parameters

While manual hemocytometry remains a reference, modern andrology employs more precise and efficient tools. Fluorescence-based automated systems (for example, NucleoCounter^®^) provide rapid, user-independent cell counts ideal for high-throughput settings by quantifying DNA-specific stains [239]. Disposable counting chambers with standardized geometries (for example, Leja^®^ slides) minimize procedural error and improve inter-laboratory consistency for concentration and motility analysis [240]. For morphology, computer-assisted sperm morphometry analysis (CASMA) systems now offer objective, high-throughput classification of sperm head dimensions and midpiece/tail defects, moving beyond subjective visual scoring [241].

### 12.2. Functional Assessment of Sperm DNA Integrity

The integrity of paternal DNA is a paramount determinant of embryonic development and pregnancy success [156,157]. Several validated assays are central to this evaluation.

#### 12.2.1. Sperm Chromatin Structure Assay (SCSA^®^)

This flow cytometric assay measures the susceptibility of sperm DNA to acid denaturation, calculating a DNA fragmentation index (DFI). A DFI > 25–30% is strongly associated with reduced conception rates and increased pregnancy loss in cattle and other species [242].

#### 12.2.2. Terminal Deoxynucleotidyl Transferase dUTP Nick End Labeling (TUNEL) Assay

This assay directly labels DNA strand breaks. Flow-cytometric TUNEL is highly sensitive, with thresholds >15–20% fragmentation indicating significant fertility impairment [243].

#### 12.2.3. Sperm Chromatin Dispersion (SCD) Test

This is a practical, microscopy-based method in which sperm with intact DNA produce characteristic halos of dispersed chromatin loops after protein removal, while fragmented DNA shows minimal dispersion [244].

#### 12.2.4. Comet Assay (Single-Cell Gel Electrophoresis)

This sensitive technique detects single and double-strand breaks at the level of individual sperm cells, visualizing fragmented DNA migrating from the nucleus to form a “comet tail” [245].

### 12.3. Omics Technologies and Genomic Selection

High-throughput “omics” technologies are identifying novel biomarkers and refining genetic selection [6,158,163].

#### 12.3.1. Proteomics and Metabolomics

Analysis of seminal plasma reveals protein and metabolic signatures correlated with fertility. In bulls, specific seminal plasma proteins, such as osteopontin, are linked to sperm function, while in boars, distinct metabolic profiles correlate with semen freezability [246,247].

#### 12.3.2. Genomics

Genomic estimated breeding values (GEBVs) for fertility traits (such as, the daughter pregnancy rate in dairy cattle) have significantly improved the accuracy of selecting high-fertility sires, even for low-heritability traits [248]. Furthermore, sequencing studies identify genetic markers and mutations associated with spermatogenic failure in livestock [249].

#### 12.3.3. Transcriptomics and Epigenetics

Analysis of spermatozoal RNA content (transcriptome) and epigenetic marks (for example, DNA methylation) provides insights into spermatogenesis, sperm function, and potential impacts on early embryogenesis, offering new layers of diagnostic information [250].

### 12.4. Synthesis and Clinical Integration

The future of male fertility assessment lies in multi-parametric, integrative approaches. No single assay is universally predictive; rather, combining conventional analysis (motility and morphology) with functional tests (DNA integrity and capacitation status) and molecular biomarkers provides a robust fertility prognosis. This integrated diagnostic panel is essential for addressing idiopathic subfertility, improving semen cryopreservation outcomes, and maximizing the genetic potential of elite sires through precise selection [31,61,170,187].

Finally, the integrative, multi-parametric approaches to male fertility assessment discussed above are built upon a wide body of foundational and specialized studies that underpin the methodologies presented throughout this review. The historical development of semen analysis, from early visual inspection [251] to the establishment of standardized laboratory parameters [252], has been crucial. Detailed assessments of sperm characteristics, including the acrosome reaction [253], specific abnormal morphologies [73,254], and the structural evaluation of bovine spermatozoa [255], have refined our understanding of male fertility. The importance of standardized protocols is further emphasized by the WHO’s laboratory manual [256], which provides a benchmark for many techniques, while the physical and chemical properties of semen itself remain a key consideration for extender design [257]. Furthermore, the assessment of female reproductive performance relies on a set of well-established indices. Equations for calculating parameters such as twinning rate, fertility, and fecundity [258,259], as well as conception rate [260] and early pregnancy assessment [261], are essential for evaluating herd productivity. Metrics like days open [262] are critical for dairy management, and understanding the factors affecting rearing [263,264] contributes to overall economic efficiency. These reproductive parameters are intrinsically linked to species-specific physiological traits, including age at puberty and gestation length, which have been extensively characterized across livestock such as cattle [265,266], buffalo [267], camels [268], goats [258,259], sheep [269], pigs [270,271], and horses [272,273]. The validation of sperm functional integrity tests, such as the hypo-osmotic swelling test, relies on foundational studies that established their relationship to other semen characteristics [96]. Similarly, the assessment of acrosomal integrity and capacitation status, as evaluated through staining techniques [102], and the application of strict morphological criteria for sperm evaluation [82,83] are grounded in this body of work. The observation of premature capacitation in cryopreserved bovine spermatozoa [76] further underscores the importance of these foundational assessments. Finally, advanced diagnostic applications, such as the use of computer-assisted semen analysis [182] and specialized techniques for cases of immotile cilia syndrome [99], demonstrate the continued evolution of the field. Collectively, the current review provides essential context and validation for the advanced techniques and comparative analyses presented, underscoring that effective fertility assessment requires integrating multiple assays tailored to specific species and production contexts.

## 13. Conclusions

This review provides a systematic examination of fertility and semen assessment methodologies in cattle, buffalo, sheep, goats, and pigs, tracing the progression from foundational morphological evaluations to sophisticated molecular and computer-assisted techniques. The synthesized evidence carries several practical implications: no single assay perfectly predicts fertility; rather, multi-parametric approaches combining conventional analysis with functional tests provide the most robust prognosis. CASA systems offer objective kinematic data but face accessibility limitations in resource-limited settings, while simple tests like HOS and eosin-nigrosin staining remain valuable in field conditions. Female fertility assessment should integrate performance metrics with an understanding of uterine health and endocrine function. Several key messages emerge from this review. Methodological evolution has progressed from subjective manual evaluation to objective and molecular approaches, yet foundational techniques remain essential reference standards. DNA integrity assessments provide prognostic value beyond conventional semen analysis, demonstrating strong correlations with fertility outcomes. Species-specific differences necessitate validation of techniques across livestock species. Integration of omics approaches is identifying novel fertility biomarkers and enabling more accurate genetic selection. Priority areas for future research include standardization of advanced assays across species, development of portable point-of-care technologies, identification of predictive biomarkers, integration of multi-omics data through systems biology, and translational research bridging in vitro findings with in vivo outcomes. Continued research and innovation remain essential to develop accessible, accurate tools that enhance reproductive efficiency in livestock production worldwide.

## Figures and Tables

**Table 1:** Analysis of academic publications on fertility measurement techniques used in this review.

Platform	Total Studies	Year Range	Dominant Topics
Elsevier	196	1945–2025	Semen cryopreservation, sperm DNA fragmentation, capacitation mechanisms, testicular morphometrics, AI-assisted sperm detection (STAR method), and epigenetic influences
Springer	46	1977–2025	Sperm motility analysis, epididymal function, follicular development, embryo transfer efficiency, and socioeconomic impacts on fertility access
Taylor & Francis	27	1985–2025	Sperm chromatin structure, oxidative stress impacts, CASA validation, and intrauterine insemination outcomes
MDPI	25	2011–2025	Sperm epigenetics, lifestyle/environmental effects, novel biomarkers (e.g., HMGA2), omics approaches (proteomics and metabolomics), and fertility biomarkers in livestock
Additional Sources	22	1938–2025	Foundational reproductive physiology, estrus synchronization, morphological studies (uterine/placental development), species-specific fertility optimization (goats, buffalo, and alpacas), and UNFPA global fertility disparity reports

## Data Availability

All data generated or analysed during this study are included in this manuscript and its Supplementary Files; (a) Supplementary File S1: S. Figures, (b) Supplementary File S2: S. Tables.

## Supplementary Materials

The following supporting information can be downloaded at: https://www.mdpi.com/article/10.3390/ani16050854/s1, (a) Supplementary file S1: Figure S1: Measuring testicular length and width using (Electronic digital callipers) for Saanen milk goat (SMG), and Dazu-black x Inner-Mongolia Cashmere crosses; Figure S2: (A) The artificial vagina (AV) for goat and sheep. (B) The artificial vagina (AV) for cattle and buffalo. Getting the semen; (C) from a male of Awassi sheep breed with a female of Awassi sheep breed in Egypt utilizing the artificial vagina (AV), (D) from male of Inner Mongolia cashmere (IMC), with a female of Dazu black (DB). (E) Comparison between A.V in rabbit “E1”, sheep & goat “E2”, and cow & buffalo “E3”. AI gun “E4”. Endoscope of sheep & goat “E5”, and cow & buffalo “E6”; Figure S3: Sperm concentration in; (A) Inner Mongolia Cashmere goat, (B) Dazu Black goat. Figure S4: Portable motility analysis system (iSperm), iSperm application on the smartphone to evaluating the percentage of sperm motility, https://www.jorvet.com/product/isperm-equine-software-w-o-ipad/ (accessed on 20 December 2025). (A) Application Interface (iSperm). (B) Tools for semen preparation, (B1) Measuring cups, (B2) Eppendorf tubes for semen samples, (B3) samples vials. (C) The final results obtained from the application; (C1) concentration 106/ml, (C2) motility (%). (D) iPad mini not with (iSperm) application (D1) Sample collector. Figure S5: A diagram shows the transformation of (MTT) to (formazan) during the reduction process in the mitochondria. (b) Supplementary file S2: Table S1: The differentiation between Oogenesis and spermatogenesis; Table S2: Common Laboratory Evaluation Techniques; Table S3: Components of Eosin-Nigrosine solution; Table S4: Components of Hypo-osmotic solutions of 150 mOsmol ml^−1^; Table S5: Components Giemsa stain and Sorensen’s phosphate buffer; Table S6: Equations used for measuring some parameters related to fertility; Table S7: The differentiation in some reproductive traits between different species.
