# Supplementary material for: A Comparative Review of Fertility and Semen Assessment Techniques in Farm Animals
Source: Animals (Basel). 2026 Mar 9;16(5):854. doi: 10.3390/ani16050854 (PMC12984749; doi:10.3390/ani16050854)
Supplement: Supplementary file 1 [file animals-16-00854-s001.zip › Supplementary File (S1); S. Figures.pdf]

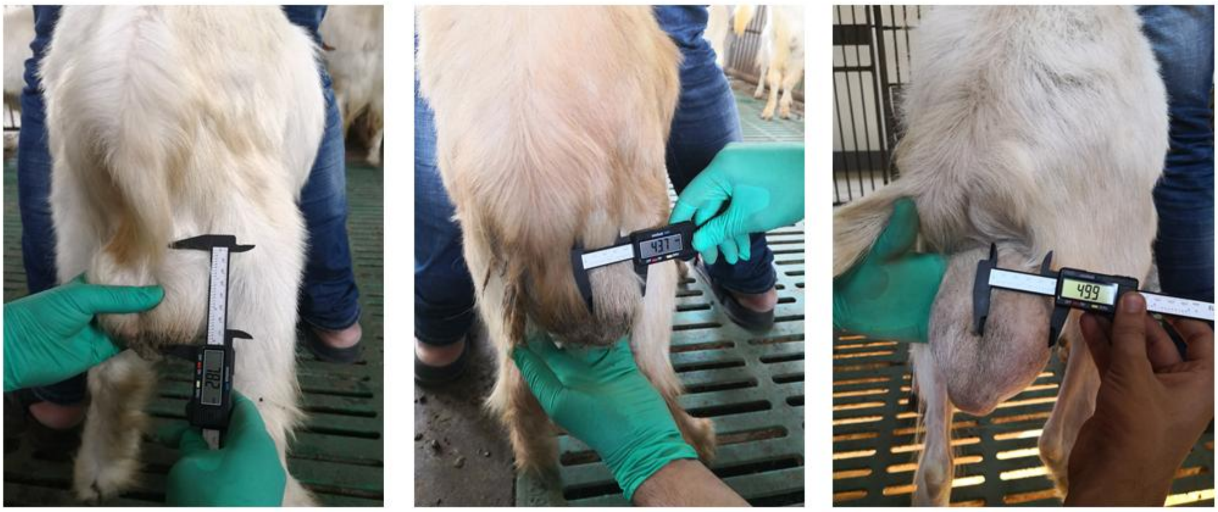

Figure S1. Measuring testicular length and width using (Electronic digital callipers) for Saanen milk goat (SMG), and Dazu-black *x* Inner-Mongolia Cashmere crosses.

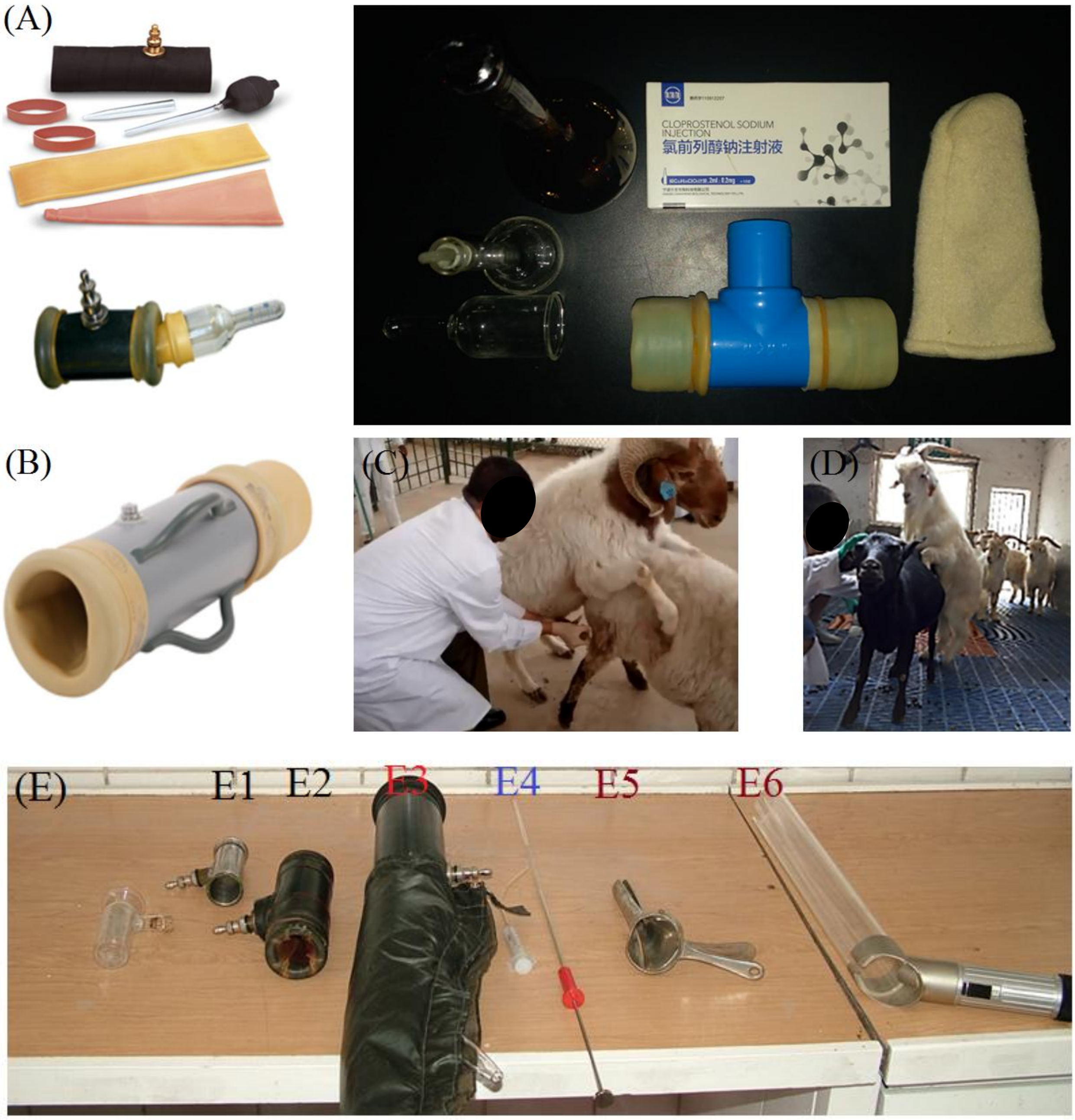

Figure S2. (A) - The artificial vagina (AV) for goat and sheep. (B) - The artificial vagina (AV) for cattle and buffalo. Getting the semen; (C)- from a male of Awassi sheep breed with a female of Awassi sheep breed in Egypt utilizing the artificial vagina (AV), (D)- from male of Inner Mongolia cashmere (IMC), with a female of Dazu black (DB). (E) - Comparison between A.V in rabbit "E1 ", sheep & goat "E2 ", and cow & buffalo "E3 ". AI gun "E4 ". Endoscope of sheep & goat "E5 ", and cow & buffalo "E6 ".

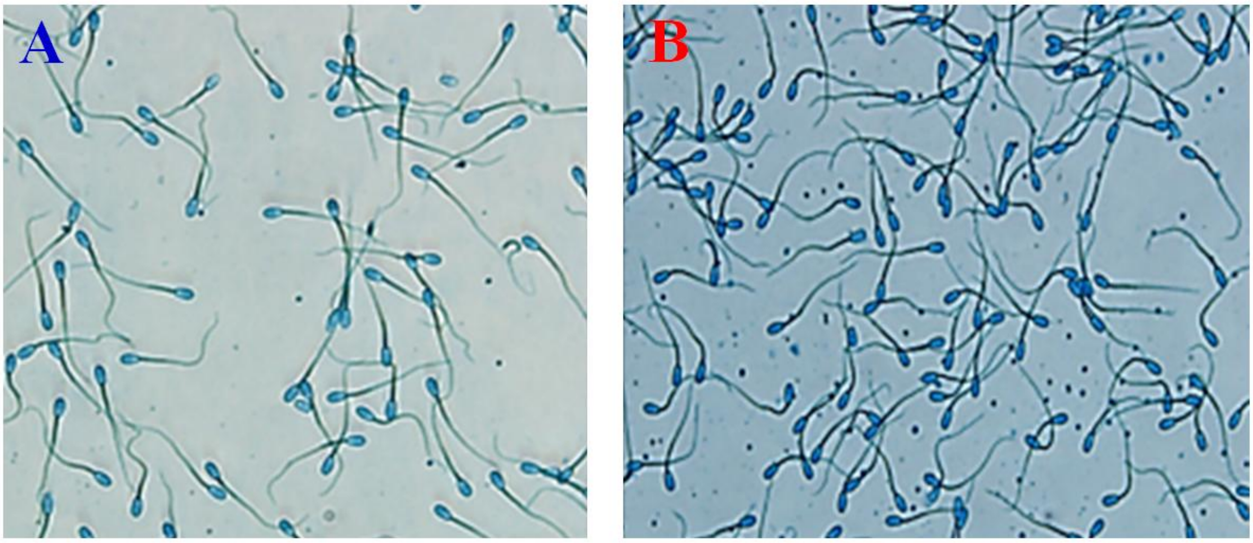

Figure S3. Sperm concentration in; (A) -Inner Mongolia Cashmere goat, (B)- Dazu Black goat.

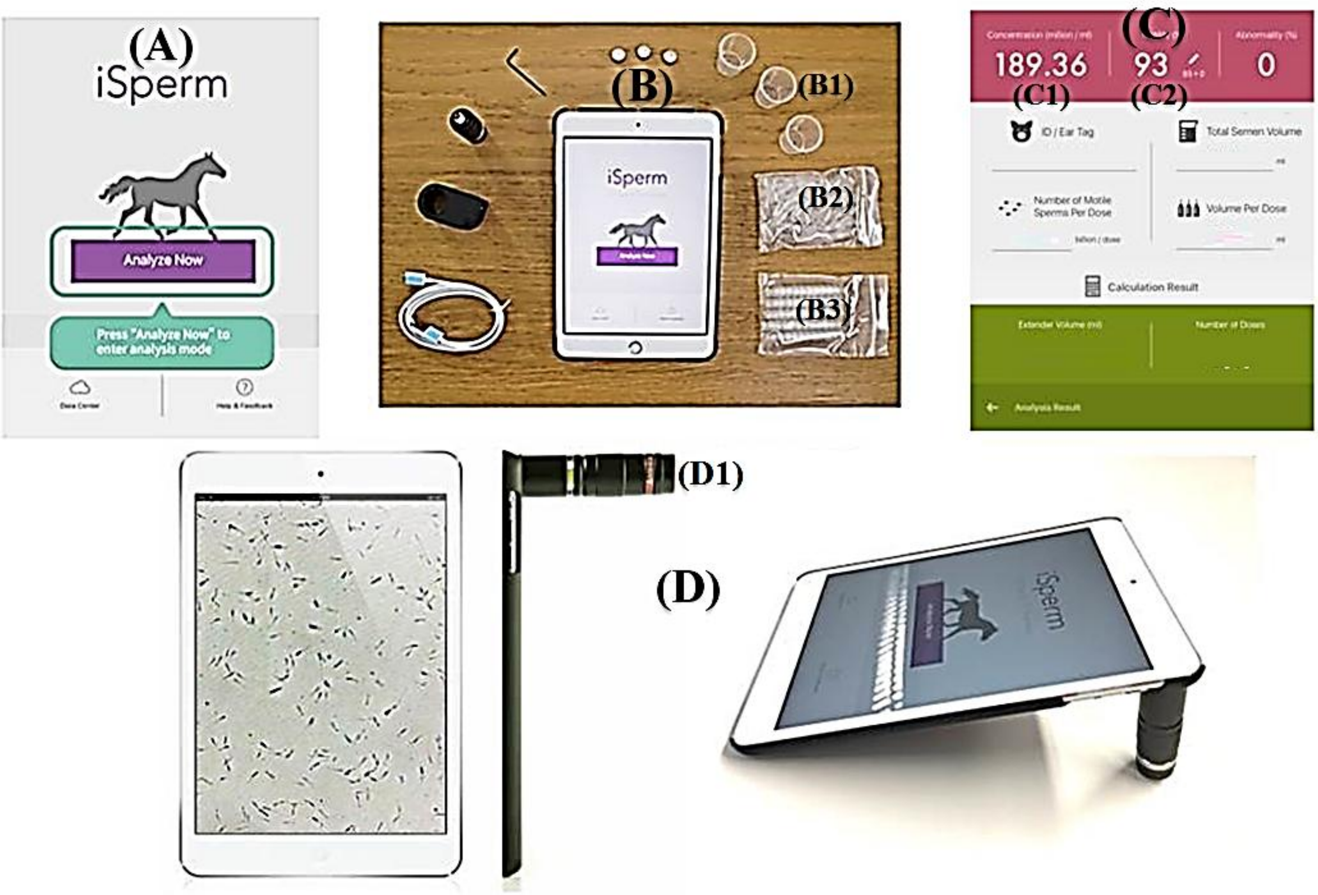

Figure S4. Portable motility analysis system (iSperm), iSperm application on the smartphone to evaluating the percentage of sperm motility, https://www.jorvet.com /product/isperm-equine-software-w-o-ipad/ (accessed on 20 December 2025). (A)- Application Interface (iSperm). (B)- Tools for semen preparation, (B_1)- Measuring cups, (B_2)- Eppendorf tubes for semen samples, (B_3)- samples vials . (C)- The final results obtained from the application; (C_1)- concentration 10^6/ml, (C_2)- motility (%). (D)- iPad mini not with (iSperm) application (D_1)- Sample collector. .

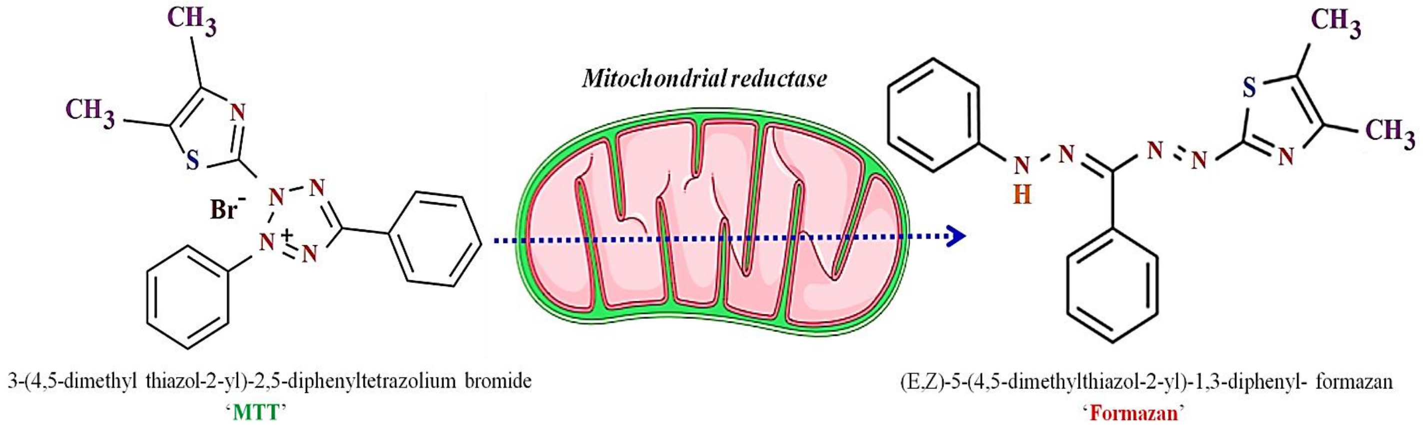

Figure S5. A diagram shows the transformation of (MTT) to (formazan) during the reduction process in the mitochondria.
