# Supplementary material for: A Comparative Review of Fertility and Semen Assessment Techniques in Farm Animals
Source: Animals (Basel). 2026 Mar 9;16(5):854. doi: 10.3390/ani16050854 (PMC12984749; doi:10.3390/ani16050854)
Supplement: Supplementary file 1 [file animals-16-00854-s001.zip › Supplementary File (S2); S. Tables.pdf]

Table S1. The differentiation between Oogenesis and spermatogenesis

Gametogensis	Oogenesis	Spermatogenesis
**The Definition**	The process that included the production of the ovum from oogonia	The process that included the production of the spermatozoa from spermatogonia
**Location**	Females` ovaries (all stages occur inside the ovary, except the last stage)	Males` testes (all stages occur inside the testis)
**Onset**	Onset of puberty	The fifth gestational week in human
**Accessory components**	□ Corona radiate<br>□ Theca folliculi<br>□ Zona pellucida<br>□ Granulosa cells	Acrosome tail
**Hormones involved**	GnRH, FSH, LH, Estrogen	GnRH, FSH, LH, Testosterone
**Motility**	Produces; immotile	Produces ;there is a motility
**Genotype (Chromosomes)**	Chromosomes + X	Chromosomes + X or Y
**Starting Cell**	Starts from a primary oocyte	Starts from a primary spermatocyte
**Number of functional cells produced**	Single ovum	Four functional spermatozoa
**Growth phase**	Too short	Prolonged
**Cytogenesis**	Two highly unequal cells	Two equal cells

Table S2. Common Laboratory Evaluation Techniques

No.	Parameters	Techniques/ Test	Ref.
1	**Colour**	White, Creamy, Milky, and Lemon colour/Visual vision	[1]
2	**Volume (ML)**	Read from a semen collection vial that contains 15 ml of graduated sterile tubes.	[2]
3	**Mass activity**	➢ A drop of thawed semen sample is uniformly spread over a clean microscope slide, warmed to 37 ºC using a thermostatic plate.<br>➢ Sperm motility is assessed under low power (10x magnification) by observing the mass movement of the spermatozoa. The evaluation of motility is based on the progressive movement of the sperm cells.	[3]
4	**Total Motility**	➢ Microscopic-Test & CASA	[4]
5	**Progressive Motility**		
6	**Sperm concentration (ML^{-9})**	➢ Currently, the spectrophotometer is the most commonly used method for analyzing spermatozoa cell concentration, due to its speed, simplicity, and reliability.<br>➢ To conduct an evaluation, a microcuvette or cuvette is filled with a diluted ejaculated sample and inserted into the spectrophotometer, where the optical density of the sample is measured. The sperm cell concentration is subsequently calculated based on the optical density readings.<br>➢ In contrast, the haemocytometer is regarded as an excellent standard for assessing spermatozoa counts.	[5]

7	**Incubation Test**	➢ The frozen semen sample straw is carefully removed from liquid nitrogen, shaken, and placed into a thawing unit set at 37°C.<br>➢ After one minute, the straw is examined for post-thaw motility using a microscope. The remaining straw is kept in the thawing unit for one hour, after which motility is assessed again using the same method. It is essential to maintain the temperature at 37°C throughout the process.	[6]
8	**Sperm viability**	Eosin-Nigrosine test	[7, 8]
9	**Morphological assessment**		[9, 10]
10	**Acrosome integrity**		[11]
11	**Sperm vitality**	Hypo-osmotic swelling test (HOST)	[12])
12	**pH**	pH paper	[13]<br>[14]

Table S3. Components of Eosin-Nigrosine solution

Component	Concentration 1	Concentration 2
Eosin-Y Yellow	0.67 g	3.3
Sodium chloride	0.9 g	1.5 g
Distilled water	100 ml	300 ml
Nigrosine	10 g	20 g
pH	7	

Table S4. Components of Hypo-osmotic solutions of 150 mOsmol ml-1

**Component**	
**Sodium citrate (g)**	0.735
**Fructose (g)**	1.351
**Millipore water (ml)**	100
**Osmolality (mOsm Kg^{-1})**	150

Table S5. Components Giemsa stain and Sorensen's phosphate buffer

Component	Concentration
**Giemsa stain**	3.8 gm
**Absolute alcohol (GR grade)**	375 ml
**Glycerol (AR grade)**	125 ml
**Preparation of Soreson`s phosphate buffer:**	
**Solution A:**	
**Sodium phosphate dibasic ($Na_2HPO_4.2H_2O$)**	11.876 gm
**Distilled water**	1000 ml
**Solution B:**	
**Potassium phosphate monobasic ($KH_2PO_4.2H_2O$)**	9.08 gm
**Distilled water**	1000 ml

Table S6. Equations used for measuring some parameters related to fertility

No.	Indices	Equations	Ref
**1**	Twining rate	$= \frac{\text{No. of twining birth}}{\text{No. of females give birth}} \text{ x } 100$	[15]
**2**	Fertility	$= \frac{\text{No. of females give birth}}{\text{Total no. of female inseminated by a male}} \text{ x } 100$	
**3**	Fecundity	$= \frac{\text{Total numbers of kids}}{\text{No. of females give birth}} \text{ x } 100$	
**4**	Kidmortality rate (%)	$= \frac{\text{Number of kids died before weaning}}{\text{Total number of kids born}} \text{ x } 100$	[16]
**5**	Services per pregnancy (Conception)	$= \frac{\text{Total matings to pregnant individuals}}{\text{Total number of pregnant individuals}}$	[17]
**6**	Pregnancy Interval (PI)	$= \frac{\text{Number of months from previous to current pregnant}}{\text{Number of individuals in the pregnancy (numerator) group}}$	[18]
**7**	Days open (OD)	$= \frac{\text{(total days from pregnancy to conception for pregnant individuals} + \text{Days from pregnancy to current date for opened individuals)}}{\text{Number of breeding individuals in the herd}}$	[19]
**8**	Birthing percentage	$\frac{\text{Number of kids born alive}}{\text{Number of kids put to the tup}} \; X \; 100$	[20]
**9**	Rearing percentage	$\frac{\text{Number of individuals reared} *}{\text{Number of females put to the tup}} \; X \; 100$	[21]

* reared = sold as finished or store kids, or sold/retained for breeding

Table S7. The differentiation in some reproductive traits between different species

Species	Age at puberty (Mo)	Age at sexual maturity (Mo)	Length of estrus cycle (d)	Duration of pregnancy (d)	Open days (d)	Litter size	*Ref.*
**Cow**	6~10	14~18	18~24	270	60~80	2.02 ± 1.02	[22]
**Buffalo**	8~12	16~20	18~24	300	60~90	2.71 ± 1.03	[23]
**Camel**	36	48	28	390	90	1	[24]
**Goat**	8~12	12~18	17~19	148	50~60	1.00~2.37	[25]
**Ewe**	8~12	12~18	16~17	148	50~60	1.00~2.81	[26]
**Sow**	2~3	3.16	21	115	***	10~14	[27]
**Mare**	9 ~10	12 ~15	21	330~345	***	***	[28]
**Human female**	120~132	***	28	270	***	***	[29]

References

1. Anderson, J., The semen of animals and its use for artificial insemination. The semen of animals and its use for artificial insemination., 1945.
2. den Daas, N., Laboratory assessment of semen characteristics. Animal Reproduction Science, 1992. 28(1-4): p. 87-94.
3. Brucker, C. and G. Lipford, The human sperm acrosome reaction: physiology and regulatory mechanisms. An update. Human reproduction update, 1995. 1(1): p. 51-62.
4. Talarczyk-Desole, J., et al., Manual vs. computer-assisted sperm analysis: can CASA replace manual assessment of human semen in clinical practice? Ginekologia polska, 2017. 88(2): p. 56-60.
5. Jeyendran, R., et al., Development of an assay to assess the functional integrity of the human sperm membrane and its relationship to other semen characteristics. Reproduction, 1984. 70(1): p. 219-228.
6. Organization, W.H., WHO laboratory manual for the examination and processing of human semen. 2010.
7. Barth, A.D. and R. Oko, Abnormal morphology of bovine spermatozoa. 1989.
8. CORMIER, N., M.A. SIRARD, and J.L. BAILEY, Premature capacitation of bovine spermatozoa is initiated by cryopreservation. Journal of Andrology, 1997. 18(4): p. 461-468.
9. Eggert-Kruse, W., et al., Clinical relevance of sperm morphology assessment using strict criteria and relationship with sperm-mucus interaction in vivo and in vitro. Fertility and sterility, 1995. 63(3): p. 612-624.

10. Van Waart, J., et al., Predictive value of normal sperm morphology in intrauterine insemination (IUI): a structured literature review. Human Reproduction Update, 2001. 7(5): p. 495-500.

11. Spindler, R., et al., Acrosomal integrity and capacitation are not influenced by sperm cryopreservation in the giant panda. Reproduction, 2004. 127(5): p. 547-556.

12. Peeraer, K., et al., Pregnancy after ICSI with ejaculated immotile spermatozoa from a patient with immotile cilia syndrome: a case report and review of the literature. Reproductive biomedicine online, 2004. 9(6): p. 659-663.

13. Owen, D.H. and D.F. Katz, A review of the physical and chemical properties of human semen and the formulation of a semen simulant. Journal of andrology, 2005. 26(4): p. 459-469.

14. Rintala, M., et al., Detection of high-risk HPV DNA in semen and its association with the quality of semen. International journal of STD & AIDS, 2004. 15(11): p. 740-743.

15. Hamd, R., et al., A study of some factors affecting fertility, fecundity and twining rate in local and Cyprus goats. Al-Anbar Journal of Veterinary Sciences, 2016. 9(2): p. 94-99.

16. Snyman, M., Factors affecting pre-weaning kid mortality in South African Angora goats. South African Journal of Animal Science, 2010. 40(1).

17. Mares, S., et al., Genetic Factoes Affecting Conception Rate and Early

Pregnancy Loss in Holstein Cattle. Journal of Dairy Science, 1961. 44(1): p. 96-103.

18. Daya, S., et al., Early pregnancy assessment with transvaginal ultrasound scanning. CMAJ: Canadian Medical Association Journal, 1991. 144(4): p. 441.
19. Dematawewa, C. and P. Berger, Genetic and phenotypic parameters for 305-day yield, fertility, and survival in Holsteins. Journal of dairy science, 1998. 81(10): p. 2700-2709.
20. Tanner, J.M., R. Whitehouse, and M. Takaishi, Standards from birth to maturity for height, weight, height velocity, and weight velocity: British children, 1965. I. Archives of disease in childhood, 1966. 41(219): p. 454.
21. Pirlo, G., F. Miglior, and M. Speroni, Effect of age at first calving on production traits and on difference between milk yield returns and rearing costs in Italian Holsteins. Journal of dairy science, 2000. 83(3): p. 603-608.
22. Bryan, M., M. Socha, and D. Tomlinson, Supplementing intensively grazed late-gestation and early-lactation dairy cattle with chromium. Journal of dairy science, 2004. 87(12): p. 4269-4277.
23. Abdalla, E., Improving the reproductive performance of Egyptian buffalo cows by changing the management system. Animal reproduction science, 2003. 75(1-2): p. 1-8.
24. Kaufmann, B.A., Reproductive performance of camels (Camelus dromedarius) under pastoral management and its influence on herd development. Livestock Production Science, 2005. 92(1): p. 17-29.

25. Norman, H., et al., Genetic and environmental factors that affect gestation length in dairy cattle. Journal of dairy science, 2009. 92(5): p. 2259-2269.
26. Rydhmer, L., Genetics of sow reproduction, including puberty, oestrus, pregnancy, farrowing and lactation. Livestock Production Science, 2000. 66(1): p. 1-12.
27. Trillmich, F., et al., Age at maturity in cavies and guinea-pigs (Cavia aperea and Cavia aperea f. porcellus): influence of social factors. Journal of Zoology, 2006. 268(3): p. 285-294.
28. Sigler, D., et al., Reproductive traits, lactation and foal growth in mares fed altrenogest. Journal of animal science, 1989. 67(5): p. 1154-1159.
29. Hurnik, J., Sexual behavior of female domestic mammals. Veterinary Clinics of North America: Food Animal Practice, 1987. 3(2): p. 423-461.
